# A modeling study to estimate prostate cancer‐specific mortality on active surveillance for men with favorable intermediate‐risk prostate cancer: Results from the SEARCH cohort

**DOI:** 10.1002/cam4.5805

**Published:** 2023-04-09

**Authors:** Paige K. Kuhlmann, Taofik Oyekunle, Zachary Klaassen, Christopher L. Amling, William J. Aronson, Matthew R. Cooperberg, Christopher J. Kane, Martha K. Terris, Stephen J. Freedland

**Affiliations:** ^1^ Division of Urology, Department of Surgery, Cedars‐Sinai Medical Center Los Angeles California USA; ^2^ Section of Urology, Durham VA Medical Center Durham North Carolina USA; ^3^ Department of Biostatistics and Bioinformatics Duke Cancer Institute, Duke University School of Medicine Durham North Carolina USA; ^4^ Department of Surgery, Section of Urology Augusta University ‐ Medical College of Georgia Augusta Georgia USA; ^5^ Department of Urology Oregon Health and Science University School of Medicine Portland Oregon USA; ^6^ Department of Urology University of California Los Angeles California USA; ^7^ Wadsworth VA Medical Center Los Angeles California USA; ^8^ Department of Urology University of California San Francisco California USA; ^9^ Department of Urology University of California San Diego California USA; ^10^ San Diego Healthcare System San Diego California USA; ^11^ Section of Urology Charlie Norwood VA Medical Center Augusta Georgia USA

**Keywords:** active surveillance, favorable intermediate risk, prostate cancer, prostate cancer‐specific mortality

## Abstract

**Purpose:**

Limited data exist to help surgeons decide between active surveillance (AS) versus treatment for men with favorable intermediate risk (FIR) prostate cancer. To estimate the theoretical excess risk of prostate cancer‐specific mortality (PCSM) with AS versus radical prostatectomy (RP), we determined the risk of PCSM in FIR men undergoing RP and modeled the PCSM risk for AS using a range of increased PSCM scenarios ranging from 1.25x to 2x higher relative to RP.

**Materials and Methods:**

We retrospectively reviewed data from men undergoing RP from 1988 to 2017 at 8 Veterans Affairs hospitals within the SEARCH cohort. Men with FIR PC were identified using the NCCN risk criteria. Risk of PCSM at 5, 10, and 15 years after RP was estimated. Using these estimates, PCSM was then modeled for AS using a range of increased risk of PCSM relative to RP ranging from 1.25x to 2x higher.

**Results:**

For the 920 FIR men identified, 5‐, 10‐, and 15‐year survival estimates for PCSM after RP were 99.9%, 99.0%, and 97.8%, respectively. If the risk of PCSM on AS were 1.25–2x greater than RP, there would be 0.54%–2.17% excess risk of PCSM at 15 years.

**Conclusions:**

The risk of death for FIR after RP is very low. Assuming even modestly increased PCSM with AS versus RP, the excess risk of death for AS in FIR is low even up to 15 years. These data support the consideration of AS as a relatively safe alternative to RP in FIR men, though prospective randomized trials are needed to validate these findings.

## Graphical abstract

The risk of death for patients with favorable intermediate risk prostate cancer after undergoing radical prostatectomy is very low. Even if we assume that active surveillance confers double the risk of prostate cancer‐specific mortality compared to surgery for this patient population, the excess risk of death for active surveillance is low. These data support the consideration of active surveillance as a relatively safe alternative to radical prostatectomy in men with favorable intermediate risk prostate cancer.

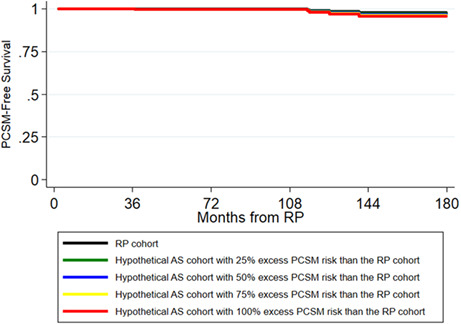

## 1 INTRODUCTION

According to most major urologic guidelines, active surveillance (AS) is the preferred option for the initial management of the majority of men with very low‐ and low‐risk prostate cancer.^1^, ^2^, ^3^, ^4^, ^5^

The use of AS for men with favorable intermediate risk (FIR) disease is less clear. According to the American Urological Association (AUA) guidelines, AS remains an option for these men, but they have a higher risk of developing metastasis compared to the lower‐risk groups.^4^ While the ProtecT trial, a randomized trial of active monitoring (AM) versus surgery versus radiation, found no difference in long‐term survival at 12 years and included men with FIR disease, the majority of men were low risk.^6^ Data on the natural history and prostate cancer‐specific mortality (PCSM) of men on AS with FIR prostate cancer is sparse, as definitive treatment has been the traditional standard of care. However, definitive treatments for prostate cancer are associated with side effects detrimental to quality of life.^7^, ^8^ Expanding inclusion criteria for AS candidacy has the potential to mitigate the substantial consequences of initial definitive treatment.

To bolster the relative dearth of information on the safety of AS in men with FIR prostate cancer, we used long‐term post‐prostatectomy data from the SEARCH cohort to estimate the risk of PCSM after surgery. Using a modeling approach, we then modeled the excess risk of PCSM conferred by AS compared to surgery in this cohort under the assumption that AS was associated with either a 1.25x, 1.5x, 1.75x, or 2x excess risk of PCSM versus surgery.

## 2 METHODS

**FIGURE 1:**
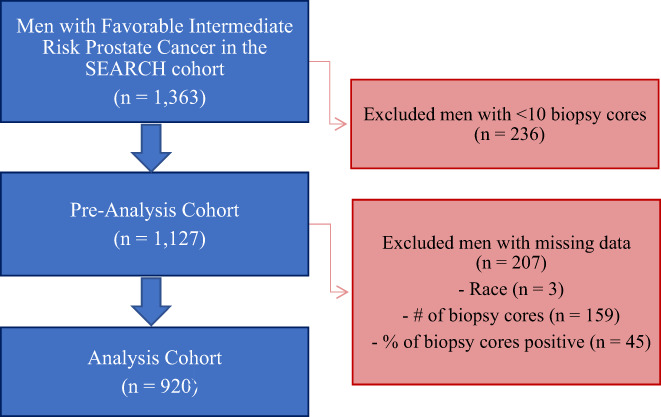
Consort diagram. The SEARCH cohort is comprised of men treated at one of eight Veterans Affairs hospitals (West Los Angeles, Palo Alto, San Francisco, Augusta, Durham, San Diego, Asheville, Portland). Favorable intermediate‐risk prostate cancer was defined using NCCN risk criteria: less than 50% of biopsy cores positive for cancer AND one of the following: clinical tumor stage cT2b to cT2c OR grade group 2 disease on biopsy (Gleason score 3 + 4 = 7) OR PSA 10–20 ng/mL.

After obtaining Institutional Review Board approval, we retrospectively reviewed all men undergoing radical prostatectomy (RP) from 1988 to 2017 at one of eight Veterans Affairs hospitals included in the Shared Equal Access Regional Cancer Hospital (SEARCH) cohort.^9^ There were 1363 (17%) men identified who met inclusion criteria for the FIR group as defined by the National Comprehensive Cancer Network (NCCN) risk criteria (less than 50% of biopsy cores positive for cancer AND one of the following: clinical tumor stage cT2b to cT2c OR grade group 2 disease on biopsy (Gleason score 3 + 4 = 7) OR PSA 10–20 ng/mL).^2^ (Figure 1) We then excluded 236 men with <10 biopsy cores. Further, men with missing data on race (*n* = 3), number of biopsy cores (*n* = 159), and percent of positive cores (*n* = 45) were excluded (Figure 1) resulting in a study population of 920 men.

The primary outcome was PCSM. The risk of PCSM after RP was estimated at 5, 10, and 15 years post‐prostatectomy with actuarial mortality data using Kaplan–Meier methods. Using these estimates, we sought to model the risk of PCSM in a comparable hypothetical cohort of men on AS. This group was assumed to be similar to the RP cohort in demographic and clinical characteristics and only differed in the choice of treatment. While it is possible PCSM with AS may be identical to surgery this would result in no excess mortality. As such, to model the possible excess mortality, for the purposes of our modeling analysis, we considered that AS was associated with a range of increased risk of PCSM relative to RP, ranging from 1.25x higher risk to 2x higher risk. Based upon the excess risk, we then calculated the number needed to harm for PCSM at 5, 10, and 15 years for our AS cohort relative to the RP cohort. Importantly, given the limited long‐term data on AS in men with FIR disease, our cohort of AS patients were hypothetical.

This analysis was repeated for secondary outcomes: castration‐resistant prostate cancer (CRPC) and development of metastasis.

Analyses were conducted using SAS 9.4 (SAS Institute) and Stata 14.2 (Stata Corp.). Statistical significance tests were two‐sided with *p* < 0.05.

## 3 RESULTS

### 3.1 Demographics

Among men in the FIR RP cohort, median (25th, 75th percentile) age was 63 (59–67) years, PSA 6.0 (4.7–8.5) ng/mL, and percent of positive biopsy core 25.0 (16.7–33.3)%. Most men were non‐black (69%), had PSA <10 ng/mL (85%), and clinical stage T1 (66%). A total of 707 (77%) men had grade group 2 indicating that this was the predominant reason these men were classified as FIR. (Table 1).

**TABLE 1:** Descriptive analysis of patient cohort.

Factors	
Age, *Median (Q1, Q3)*	63 (59, 67)
Race, *n (%)*	
Black	286 (31%)
Non‐black	634 (69%)
PSA (ng/mL), *Median (Q1, Q3)*	6.0 (4.7, 8.5)
PSA category, n (%)	
<10 ng/mL	771 (85%)
≥10 ng/mL	149 (15%)
Pre‐op grade group, *n (%)*	
1	213 (23%)
2	707 (77%)
Percent of biopsy core positive (%), *Median (Q1, Q3)*	25 (17, 33)
Clinical stage, *n (%)*	
T1	608 (66%)
T2a	248 (27%)
T2b	33 (4%)
T2c	31 (3%)
Year of surgery, *Median (Q1, Q3)*	2011 (2008, 2014)
Surgery center, *n (%)*	
West LA	114 (12%)
Palo Alto	80 (9%)
San Francisco	120 (13%)
Augusta	170 (19%)
Durham	131 (14%)
San Diego	120 (13%)
Asheville	58 (6%)
Portland	127 (14%)

Abbreviations: Q1, 25th percentile; Q3, 75th percentile.

### 3.2 Risk of various outcomes in the RP cohort

For men undergoing RP, survival estimates for PCSM were 99.9% at 5 years, 99.0% at 10 years, and 97.8% at 15 years (Table 2). The estimated probabilities of CRPC‐free and metastasis‐free survival were 99.8% and 99.3% at 5 years, 97.5% and 97.1% at 10 years, and 96.6% and 96.5% at 15 years, respectively (Table 2).

**TABLE 2:** 5‐, 10‐, and 15‐year survival estimates (%) and 95% CI for favorable intermediate‐risk group in the radical prostatectomy (RP) cohort.

Time points	Survival estimates (%), (95% confidence intervals)		
	PC‐specific survival	CRPC‐free survival	Metastasis‐free survival
5‐years	99.9 (99.0–99.9)	99.8 (98.9–100.0)	99.3 (98.4–99.7)
10‐years	99.0 (96.7–100.0)	97.5 (94.9–98.8)	97.1 (94.8–98.4)
15‐years	97.8 (94.5–99.1)	96.6 (93.1–98.4)	96.5 (93.8–98.1)

Abbreviations: CRPC, astration resistant prostate cancer; PC, Prostate cancer.

### 3.3 Risk of various outcomes in the hypothetical AS cohort

**FIGURE 2:**
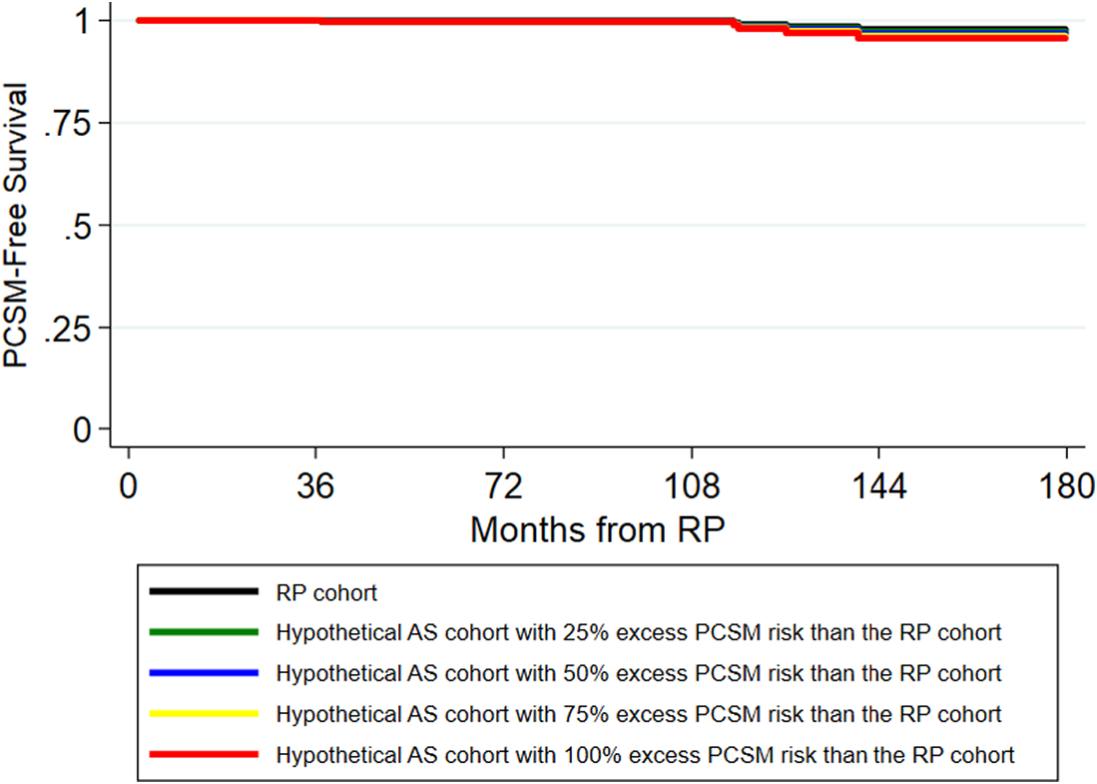
Kaplan–Meier curves of PCSM‐free survival probabilities for the RP cohort and hypothetical AS cohorts.

**FIGURE 3:**
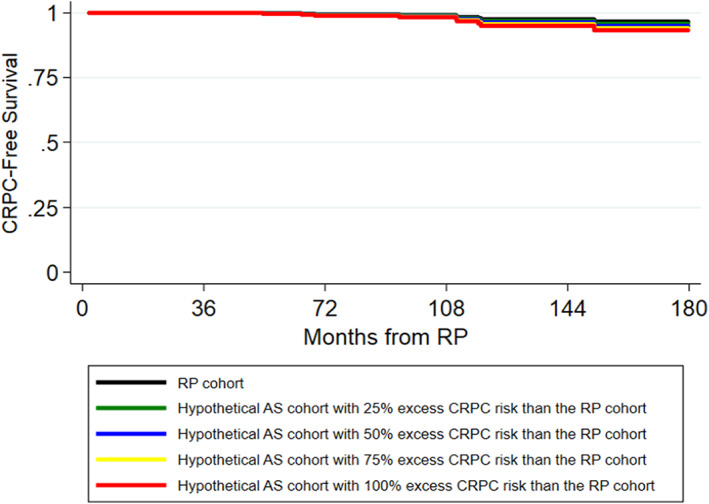
Kaplan–Meier curves of CRPC‐free survival probabilities for the RP cohort and hypothetical AS cohorts.

**FIGURE 4:**
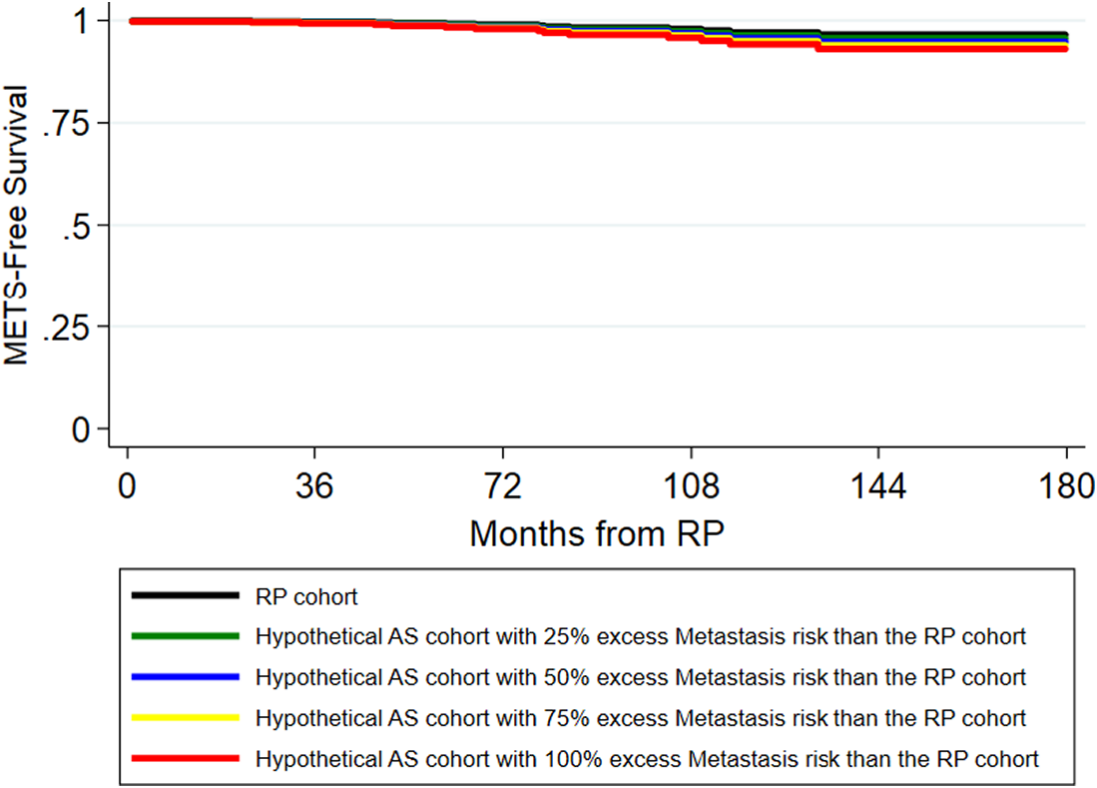
Kaplan–Meier curves of Metastasis‐free survival probabilities for the RP cohort and hypothetical AS cohorts.

Table 3 summarizes the excess risk of PCSM, CRPC, and metastasis on AS at 5, 10, and 15 years, assuming that AS carries 1.25, 1.5, 1.75, or 2 times the risk of PCSM, CRPC, and metastasis compared to RP. PCSM‐free, CRPC‐free, and metastasis‐free survival curves for the RP and hypothetical AS cohorts are depicted in Figures 2, 3, 4. As noted in the survival curves, the excess risk of adverse outcomes in the hypothetical AS cohorts is small. Specifically, under the assumption that the hypothetical AS cohort had a 1.25x higher risk of PCSM than the RP cohort, the excess risk of PCSM in the AS cohort compared to the RP cohort was small and <1% even at 15 years (Table 3). Similarly, the excess risk of CRPC and metastasis in the hypothetical AS cohort compared to the RP cohort was also <1% at 15 years.

**TABLE 3:** Hypothetical estimate of excess probability of PCSM, CRPC, and metastasis in AS group as a function of the estimated probability of PCSM, CRPC, and metastasis in the RP cohort.

Time points	Estimated probability of PCSM (x) in RP cohort	Excess PCSM risk in AS			
		1.25X	1.50X	1.75X	2.00X
5‐years	x = 0.14%	0.04%	0.07%	0.11%	0.14%
10‐years	x = 0.99%	0.25%	0.50%	0.74%	0.99%
15 years	x = 2.17%	0.54%	1.09%	1.63%	2.17%

Time points	Estimated Probability of CRPC (x) in RP cohort	Excess CRPC risk in AS			
		1.25X	1.50X	1.75X	2.00X
5‐years	x = 0.16%	0.04%	0.08%	0.12%	0.16%
10‐years	x = 2.48%	0.62%	1.24%	1.86%	2.48%
15 years	x = 3.35%	0.84%	1.68%	2.52%	3.35%

Time points	Estimated Probability of Metastasis (x) in RP cohort	Excess Metastasis risk in AS			
		1.25X	1.50X	1.75X	2.00X
5‐years	x = 0.67%	0.18%	0.34%	0.50%	0.67%
10‐years	x = 2.90%	0.73%	1.45%	2.18%	2.90%
15 years	x = 3.47%	0.87%	1.74%	2.60%	3.47%

Abbreviations: AS, active surveillance; CRPC, Castration resistant prostate cancer; PCSM, Prostate cancer‐specific mortality; RP, radical prostatectomy.

As the estimated risk of adverse outcomes relative to the RP cohort increased from 1.25x to 1.5x to 1.75x to 2x, the excess risk of adverse outcomes increased. However, even with a 2x increased risk of adverse outcomes, the excess risk of PCSM with AS remained modest and was only 2.17% at 15 years (Table 3). Likewise, the excess risk of metastasis (3.47%) and CRPC (3.35%) also remained low at 15 years.

### 3.4 Number needed to harm

Table 4 summarizes the number needed to harm (NNH) assuming AS has 1.25, 1.5, 1.75, and 2 times the risk of PCSM relative to RP at the 5‐, 10‐, and 15‐year time points. Given a 1.25x increase in the estimated PCSM in the AS cohort compared to the RP cohort, for every 184 men who initially choose AS instead of RP, there will be one excess PC death at 15 years (Table 4). As we modeled higher excess risks of PCSM up to 2x increased risk, the NNH decreased but remained modest. Specifically, NNH with 1.5x, 1.75x, and 2x risk was 92, 61, and 46, respectively, at 15 years for PCSM.

**TABLE 4:** Number needed to harm (NNH) for prostate‐specific cancer mortality (PCSM) at 5, 10, and 15 years for active surveillance (AS) compared to radical prostatectomy (RP).

Time	NNH for AS compared to RP			
	1.25x	1.50x	1.75x	2.00x
5‐years	2857	1429	952	714
10‐years	404	202	134	101
15 years	184	92	61	46

## 4 DISCUSSION

There is a paucity of data to inform the use of AS as the initial management strategy for men with FIR prostate cancer. Whether AS negatively impacts PSCM in these men is unknown. Using the SEARCH cohort, which captures long‐term outcomes after prostatectomy in a large cohort of men with prostate cancer treated at Veterans Affairs facilities, we found that the modeled excess risk of PCSM if these men had been managed with AS is low. Even if AS confers twice the risk of PCSM compared to surgery, the estimated 15‐year excess risk of PCSM is only 2%. This means that for every 46 men placed on AS instead of upfront prostatectomy, there would be only one extra prostate cancer‐related death 15 years after diagnosis. Together, these data suggest that AS is a reasonable option for men with FIR prostate cancer who are willing to accept a low, but non‐zero, potential increased risk of death in exchange for preserved quality of life.

Current guidelines vary in their recommendations regarding the use of AS for men with FIR disease. The NCCN guidelines consider AS as an appropriate option for select men with FIR disease, but emphasize the higher risk of developing metastasis compared to definitive treatment.^2^, ^10^ The Cancer Care Ontario (CCO) guidelines state that AS can be considered for patients with low‐volume, low‐percentage Gleason 4 pattern grade group 2 disease and/or men older than 75 years.^1^ The AUA/American Society for Radiation Oncology (ASTRO)/Society of Urologic Oncology (SUO) guidelines include AS as an option for men with FIR disease and an estimated life expectancy >5 years, but also emphasize informing the patient that AS has a higher risk of metastasis than definitive treatment.^3^, ^4^ The hesitation to a more confident recommendation of AS in these patients historically stems from several studies reporting worse outcomes compared to low‐risk disease in the context of management strategies other than AS. One retrospective analysis of men undergoing RP at a single institution, which included 608 with low‐volume intermediate‐risk disease and 4849 with low‐risk disease, reported a 25% rate of adverse pathologic features in the prostatectomy specimen in the FIR group compared to 6% in the low‐risk group. This study did not report on mortality outcomes.^11^ The SPCG‐4 trial assessed PCSM in 695 men with localized prostate cancer randomized to prostatectomy or watchful waiting with a median 13‐year follow‐up. Compared to the prostatectomy group, men with a low‐risk disease in the watchful waiting group had a non‐significant 4% higher absolute risk of PCSM, while men with the intermediate‐risk disease in this group had a 24% higher absolute risk of PCSM. The inclusion criteria for each risk category were not described.^12^ The PIVOT trial similarly assessed PCSM in 731 men with localized disease randomized to surgery or observation with a median follow‐up of 10 years. They found no difference in PCSM between treatment groups for men with PSA <10 ng/mL, but for men with a PSA >10 ng/mL (one of the inclusion criteria for the NCCN intermediate risk category), the 6% rate of PCSM in the surgery group was significantly lower than the 13% rate of PCSM in the observation group.^13^ The results of these and other studies led to the widespread adoption of AS for low‐risk disease, but were less convincing regarding the safety of AS for intermediate‐risk diseases.

Despite the conclusions of these studies, the theory that AS, which involves close monitoring and delayed treatment when necessary, should result in improved outcomes compared to watchful waiting/observation has led to some trialing of AS in men with intermediate‐risk disease. The only randomized trial comparing upfront treatment to AM to date is the ProtecT trial. In total, 1643 men with clinically localized prostate cancer were randomized to AM, surgery, or radiation, and then PCSM was assessed at 10 years. The AM protocol consisted of periodically checking PSA values, with increases >50% within the previous 12 months triggering a review of possible management options. Of note, this differs from modern AS, during which men undergo periodic repeat prostate biopsies +/− prostate MRI. While men on AM were 1.7 times more likely to die of prostate cancer compared to men undergoing initial treatment, this difference was not significant. Additionally, the absolute number of deaths in the total cohort was low, only 17 of 1643. While this trial did include men with intermediate‐risk disease, the majority of men had a low‐risk disease.^6^ Given most ProtecT patients were low‐risk, it is possible that in FIR, the excess risk of mortality is greater than 1.7. Alternatively, because ProtecT included high‐risk men, it is possible the high‐risk patients drove the mortality excess and it is even lower in FIR. To accommodate these dueling possibilities, we modeled excess risk ranging from 1.25x to 2x higher. One single‐institution prospective cohort study of patients with low‐ (*n* = 769) and intermediate‐risk (*n* = 211) disease managed with AS reported that PCSM at 10 and 15 years was 4.5% and 11.5% in the intermediate‐risk group, compared to 1.8% and 3.7% in the low‐risk group. However, the intermediate‐risk group included favorable and unfavorable risk patients.^14^ Using data from SEER, another group reported worse 5‐year PCSM in patients with an intermediate‐risk disease on AS compared to those with a low‐risk disease on AS, as well as those who underwent initial treatment (1.1% vs. 0.1% vs. 0.4%). However, after separating out the intermediate risk group into favorable and unfavorable, there was no difference in 5‐year PCSM between the FIR group on AS compared to low risk on AS or initial treatment.^15^ Another retrospective review of SEER data suggested that AS may be safe in men with FIR as long as the biopsy Gleason score does not go above 7.^16^ The results of our study are consistent with the numbers reported in the ProtecT trial. The 10‐year estimated prostate cancer‐specific survival after prostatectomy in our cohort was 99%, identical to that in the ProtecT trial. If we were to assume that AS confers a 1.75 times risk of PCSM compared to treatment (which is the closest value in our model to the ProtecT trial's finding of 1.7x increased risk with AM), that would result in a 0.74% increased risk of PCSM after 10 years. This equates to 1 prostate cancer‐related death for every 134 men being placed on AS instead of surgery.

The implications of expanding candidacy for AS to men with FIR prostate cancer are potentially quite impactful for patients. The option of AS gives men the chance to avoid or delay the significant side effects of upfront treatment. Patient‐reported sexual function, bowel function, urinary continence, nocturia, and other lower urinary tract symptoms were worse in one or both of the treatment groups compared to the AM group in the ProtecT trial.^6^ While oncologic outcomes are often the priority for physicians, effect on quality of life needs to be considered. When improvement in cancer‐specific survival with a given treatment is modest, even if statistically significant, its clinical significance should be weighed against the side effects that come with the treatment.

The degree of improvement in PCSM with initial treatment also needs to be contextualized with competing risks of death in this patient population. Our results suggest that if AS confers twice the risk of PCSM compared to surgery, PCSM at 10 years after diagnosis would be 1.98%. The median age of men with newly diagnosed prostate cancer is 67 years.^17^ Estimates for all‐cause 10‐year mortality for men this age range from ~17% to 40%, depending on comorbidities.^18^, ^19^ While treatment decisions must be individualized to each patient, these data taken together suggest that men with FIR disease placed on AS are much more likely to die of a cause other than prostate cancer. Furthermore, taking into account patient age and comorbidities, as well as factors like prostate MRI findings, results of genetic testing, ethnicity, and family history, may help in identifying patients within the FIR category who are more or less appropriate candidates for AS.

This study has several limitations worth mentioning. First, the use of multiple testing and multiple outcomes in our statistical analysis should be noted. Second, it is a modeling study, so the cohort of patients on AS is purely hypothetical. However, the use of multiple increased risk scenarios, which spans the reported increased risk with AS from the only randomized trial on AM versus treatment, yields a range of realistic possible outcomes. While it is possible the excess risk of death with AS is greater than 2x, the best available data from ProtecT would suggest it is less than this, though based upon small numbers of deaths in ProtecT. Additionally, these results need to be validated in a non‐Veteran population. Despite its limitations, this study contributes important information that may help guide the use of AS for men with FIR prostate cancer, a topic on which evidence is currently lacking.

## 5 CONCLUSION

In conclusion, the risk of death for patients with FIR prostate cancer after undergoing RP is very low. Even if we assume that AS confers double the risk of PSCM compared to RP for patients with FIR disease, the excess risk of death for AS is low. These data support the consideration of AS as a relatively safe alternative to RP in men with FIR prostate cancer. However, prospective randomized trials are needed to validate these findings.

## AUTHOR CONTRIBUTIONS

**Paige Kiley Kuhlmann:** Conceptualization (equal); formal analysis (equal); investigation (equal); methodology (equal); writing – original draft (equal); writing – review and editing (equal). **Taofik O Oyekunle:** Formal analysis (equal); software (equal); writing – review and editing (equal). **Zachary Klaassen:** Data curation (equal); funding acquisition (equal); investigation (equal); project administration (equal); resources (equal); writing – review and editing (equal). **Christopher L Amling:** Data curation (equal); funding acquisition (equal); project administration (equal); resources (equal); writing – review and editing (equal). **William Aronson:** Data curation (equal); funding acquisition (equal); project administration (equal); resources (equal); writing – review and editing (equal). **Matthew R. Cooperberg:** Data curation (equal); funding acquisition (equal); project administration (equal); resources (equal); writing – review and editing (equal). **Christopher J. Kane:** Data curation (equal); funding acquisition (equal); project administration (equal); resources (equal); writing – review and editing (equal). **Martha K. Terris:** Data curation (equal); funding acquisition (equal); project administration (equal); resources (equal); writing – review and editing (equal). **Stephen Freedland:** Conceptualization (equal); data curation (equal); formal analysis (equal); funding acquisition (equal); investigation (equal); methodology (equal); project administration (equal); supervision (equal); writing – original draft (equal); writing – review and editing (equal).

## FUNDING INFORMATION

There are no sources of funding to disclose for this article.

## CONFLICT OF INTEREST STATEMENT

There are no author conflicts of interest to disclose for this article.

## Data Availability

Data sharing is not applicable to this article as no new data were created or analyzed in this study.
